# Fusion of a Short Peptide that Binds Immunoglobulin G to a Recombinant Protein Substantially Increases Its Plasma Half-Life in Mice

**DOI:** 10.1371/journal.pone.0102566

**Published:** 2014-07-24

**Authors:** Jonathan T. Sockolosky, Saul Kivimäe, Francis C. Szoka

**Affiliations:** 1 Pharmaceutical Sciences and Pharmacogenomics Graduate Program, University of California San Francisco, San Francisco, California, United States of America; 2 Departments of Bioengineering, Therapeutic Sciences and Pharmaceutical Chemistry, University of California San Francisco, San Francisco, California, United States of America; Baker IDI Heart and Diabetes Institute, Australia

## Abstract

We explore a strategy to substantially increase the half-life of recombinant proteins by genetic fusion to FcIII, a 13-mer IgG-Fc domain binding peptide (IgGBP) originally identified by DeLano and co-workers at Genentech [DeLano WL, et al. (2000) *Science* 287∶1279–1283]. IgGBP fusion increases the *in vivo* half-life of proteins by enabling the fusion protein to bind serum IgG, a concept originally introduced by DeLano and co-workers in a patent but that to the best of our knowledge has never been pursued in the scientific literature. To further investigate the *in vitro* and *in vivo* properties of IgGBP fusion proteins, we fused FcIII to the C-terminus of a model fluorescent protein, monomeric Katushka (mKate). mKate-IgGBP fusions are easily expressed in *Escherichia coli* and bind specifically to human IgG with an affinity of ∼40 nM and ∼20 nM at pH 7.4 and pH 6, respectively, but not to mouse or rat IgG isotypes. mKate-IgGBP binds the Fc-domain of hIgG1 at a site overlapping the human neonatal Fc receptor (hFcRn) and as a consequence inhibits the binding of hIgG1 to hFcRn *in vitro*. High affinity binding to human IgG also endows mKate-IgGBP with a long circulation half-life of ∼8 hr in mice, a 75-fold increase compared to unmodified mKate. Thus, IgGBP fusion significantly reduces protein clearance by piggybacking on serum IgG without substantially increasing protein molecular weight due to the small size of the IgGBP. These attractive features could result in protein therapies with reduced dose frequency and improved patient compliance.

## Introduction

Protein-based therapeutic sales in the biotechnology industry continue to soar reaching ∼$64 billion in 2012, a remarkable ∼18% increase compared to 2011 [1]. Arguably monoclonal antibodies (mAbs) are the most successful class of protein drugs due to their safety, target specificity, and exceptionally long half-life. Not far behind are the rapidly growing classes of hormones, cytokines, growth factors, enzymes, and blood factors [1]. Unlike mAbs, these classes of human proteins are rapidly eliminated from circulation due to their small size, which necessitates their frequent injection or continuous infusion to maintain therapeutic blood concentrations.

To overcome these limitations a number of protein half-life extension strategies have been devised [2] including chemical conjugation [3] or genetic fusion to high molecular weight polymers [4], genetic fusion to the Fc-domain of immunoglobulin G (IgG) [5] or monomeric IgG domains [6]–[8], and genetic fusion to albumin [9]. Fc- and albumin fusion increase protein half-life by increasing the size (e.g. hydrodynamic radius) of the modified protein and in turn reducing renal clearance. In addition to increasing size, Fc- and albumin fusion enables interaction with the neonatal Fc receptor (FcRn), which salvages bound ligands from intracellular catabolism by recycling them back to the circulation [10], [11]. This interaction with FcRn contributes to the extraordinarily long, ∼21 day serum half-life of albumin and IgG in humans [12]. Therefore, engineering proteins to interact with serum IgG or albumin has the potential to significantly increase half-life by reducing both renal clearance and intracellular catabolism.

Bacterial derived IgG [13] and albumin [9] binding domains have been used as fusion partners to extend half-life; however, immunogenicity of non-human proteins is a concern. Albumin binding peptides [14] or proteins derived from human antibody domains [15] have been engineered to overcome the immune system limitations. When fused to a protein cargo, these albumin ligands substantially increase protein half-life by binding serum albumin thereby reducing the clearance of low molecular weight proteins.

**Figure 1:**
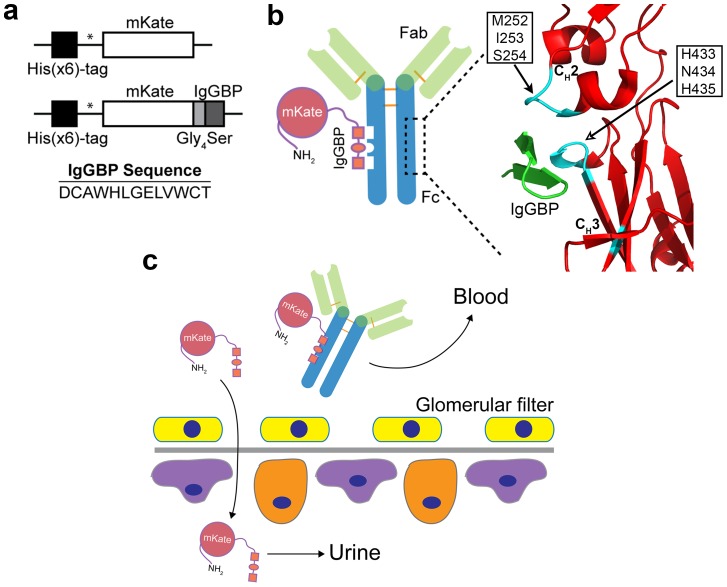
IgGBP fusion as a strategy to improve protein half-life by targeting serum IgG. (**a**) Schematic of genes encoding mKate or mKate modified at its C-terminus with an IgGBP sequence. * indicates thrombin cleavage site for removal of poly-histidine tag. (**b**) Cartoon depicting binding of mKate-IgGBP to the Fc-domain of hIgG and the corresponding crystal structure [16] of the IgGBP (green) in complex with Fc (red) (PDB 1DN2). Critical Fc amino acids that contribute to IgGBP binding at the C_H_2–C_H_3 interface are colored cyan. The same set of Fc residues is critical for FcRn binding. (**c**) Proposed half-life extension mechanism of IgGBP fusion. mKate-IgGBP binds serum IgG thus restricting its excretion through the kidney whereas unbound mKate-IgGBP is cleared through the glomerulus.

In addition to albumin binding peptides, DeLano et al. identified peptides that bind IgG by phage display [16]. One of the identified peptides, FcIII, binds with ∼20–40 nM affinity to the Fc-domain of human IgG at the interface between its C_H_2 and C_H_3 domains (Figure 1b), a binding site that is recognized by a number of other human and non-human proteins including the FcRn, staphylococcal protein A (SpA) and streptococcal protein G (SpG) [16]. In a patent, DeLano and co-workers demonstrated that FcIII fusion to a Fab fragment increases its half-life by ∼36-fold in rabbits; however, to the best of our knowledge this approach has never been evaluated the literature.

We have focused our efforts on protein engineering strategies that have the potential to enable long circulation with a minimal increase in protein molecular weight [17]. Given the low molecular weight, simple structure, and non-bacterial origin of the IgGBPs identified by DeLano, we reasoned that FcIII fusion would be a simple and attractive method to improve protein half-life by piggybacking on serum IgG (Figure 1c) and warranted further investigation.

Therefore, we built upon the Dennis and DeLano tactic to improve protein half-life by genetic fusion of the 13-mer DeLano peptide (FcIII) to the C-terminus of a model fluorescent protein (Figure 1a). We designated FcIII as the IgG-Fc binding peptide (IgGBP) when genetically fused to a protein. We demonstrate that mKate-IgGBP fusion proteins can be expressed in *E. coli*, bind with high affinity and specificity to the Fc-domain of human IgG1, and have an extended half-life in mice. These results confirm the previous findings of Dennis and DeLano et al. indicating that genetic modification of recombinant proteins with short peptides that bind abundant serum proteins, such as albumin or IgG, is a general strategy to improve protein pharmacokinetics [14], [18]. Thus, IgGBP fusion is an additional strategy to improve the half-life of recombinant proteins.

## Materials and Methods

### Materials

Human IgG1 (Avastin, bevacizumab) was obtained from the UCSF Medical Center and mouse IgG1 (MOPC-21 isotype control), mouse IgG2a (CI.18 isotype control), mouse IgG2b (MPC-11 isotype control), and rat IgG2b (LTF-2 isotype control) were obtained from the UCSF Monoclonal Antibody Core facility. Donkey anti-chicken IgY IgG-HRP was from Jackson ImmunoResearch (West Grove, PA). Ovalbumin, ampicillin, Terrific Broth (TB), 3,3′,5,5′-tetramethylbenzidine (TMB), heparin, and all buffer salts were purchased from Sigma-Aldrich (St. Louis, MO). Recombinant Protein A (SpA) was purchased from Pierce (Rockford, IL). Alexa Fluor 647 was purchased from Life Technologies (Grand Island, NY). AnaTag 5– TAMRA protein labeling kit was purchased from AnaSpec (Fremont, CA). Nickel Sepharose high performance resin prepacked in 5 mL HiTrap columns (HisTrap FF), PD-10 desalting columns, and Superdex 75 size exclusion chromatography (SEC) column were purchased from GEHealthcare (Piscataway, NJ). Complete EDTA-free protease inhibitor cocktail tablets and isopropyl β-D-1-thiogalactopyranoside (IPTG) were purchased from Roche Diagnostics (Indianapolis, IN). Steri-cup 0.45 µm vacuum filters and Amicon 10 kDa MWCO spin filters were from Millipore (Billerica, MA). All restriction enzymes and buffers used for cloning were purchased from New England Biolabs (Beverly, MA) and all primers were purchased from IDT (San Diego, CA).

### Cell lines

MDCK wild type cells (purchased from the UCSF cell culture facility) were maintained in MEM supplemented with 10% FBS, 1% non-essential amino acids (NEAA), 1% L-glutamine, 1% sodium pyruvate, and 1% penicillin and streptomycin. MDCK hFcRn-EYFP/hβ2M cells were generated as previously described [17] and maintained in MDCK wild type media supplemented with 0.3 mg/mL Hygromycin B and 0.4 mg/mL G418. All cells were maintained in a humidified environment at 37°C and 5% CO_2_.

### Mice

All mice used in this study were purchased from The Jackson Laboratory (Bar Harbor, ME), bred, and maintained under pathogen-free conditions at the University of California, San Francisco (UCSF). All breeding schemes and mouse procedures were approved by the UCSF Institutional Animal Care and Use Committee (IACUC). Three mouse strains were used in this study: control C57BL/6J (wild type; stock number 000664), homozygous B6.Cg-*Fcgrt^tm1Dcr^* Tg(FCGRT)32Dcr/DcrJ (hFcRn Tg; stock number 014565), and homozygous B6.Cg-*Fcgrt^tm1Dcr^*/DcrJ (FcRn^−/−^; stock number 003982). C57BL/6J mice are wild type at both the mouse Fcgrt and beta-2 microglobulin (mβ2m) locus resulting in a fully murine FcRn/β2m receptor. hFcRn Tg mice are knock out for mouse *Fcgrt* and express a human *FCGRT* gene under control of the human *FCGRT* promoter by insertion of a 33-Kb cosmid clone including the complete *FCGRT* gene (approximately 11 kb as well as 10 kb of 5′ and 3′ flanking sequences) [19]. The hFcRn Tg mice are wild type at the murine β2m locus and therefore express a hybrid hFcRn/mβ2m heterodimeric receptor at the protein level. FcRn^−/−^ mice are knock out for mouse Fcgrt and therefore lack FcRn at the protein level.

### *E. coli* expression vectors

The *E. coli* expression vector encoding monomeric Katushka (mKate), pET15b_mKate, was described previously [17]. The gene encoding mKate modified at its C terminus with the IgG-binding polypeptide (IgGBP) sequence was constructed by PCR amplification of mKate (from pET15b_mKate) with primers designed to insert DNA encoding the IgGBP sequence (DCAWHLGELVWCT) separated from the C-terminus of mKate by a flexible linker (GGGGS). The primer sequences are as follows: 5′-CGGCAGCCATATGTCTGAACTGATCA-3′ and 5′- GCAGCCGGATCCTTAGGTGCACCACACCAGTTCGCCCAGATGCCACGCGCAATCCGAGCCGCCGCCGCCTTTATGGCCCAGTTTAGA-3′. The resulting PCR product was restriction-cloned into the NdeI and BamHI restriction sites of the bacterial expression vector pET15b (Novagen) to yield pET15b_mKateIgGBP. The pET15b_mKateIgGBP vector encodes mKate-IgGBP containing a N-terminal poly-histidine tag for purification by immobilized metal affinity chromatography (IMAC), followed by a thrombin cleavage sequence. All PCR reactions were performed with Phusion DNA polymerase (New England BioLabs). All plasmids were confirmed by DNA sequencing.

### Protein expression and purification

Expression of mKate and C-terminal IgGBP mKate (mKate-IgGBP) was carried out in BL21-Codon Plus (DE3)-RIPL *E. coli* cells (Stratagene; La Jolla, CA) harboring the expression vectors described above. A 100 mL overnight *E. coli* culture was used to inoculate a 1 L culture of Terrific Broth containing 100 µg/mL ampicillin and 0.1 mM IPTG. Cells were cultured at 37°C for 8 hrs and harvested by centrifugation. Cells were lysed by free-thaw and lysozyme treatment (1 mg/mL) followed by sonication and centrifugation. The supernatant containing soluble proteins were purified by Ni^2+^ affinity chromatography followed by size exclusion chromatography. The N-terminal poly-histidine tag was cleaved with thrombin (Amersham Biosciences; Piscataway, NJ) and removed by Ni^2+^ affinity chromatography. Purity was confirmed by SDS-PAGE.

### Matrix-assisted laser desorption and ionization (MALDI)-time of flight (TOF) mass spectrometry

The intact mass of purified mKate and mKate-IgGBP was determined by MALDI-TOF mass spectrometry. Purified proteins were desalted using C_4_ ZipTips (Millipore; Billerica, MA) per the manufactures recommended protocol and eluted with 4 µL of 75% acetonitrile, 0.1% TFA in water. Desalted proteins (1 µL) were mixed with 1 µL of a saturated solution of sinapinic acid (SA) and spotted on top of a pre-formed layer of SA matrix. Mass spectra were obtained on a Microflex LT mass spectrometer (Bruker Daltonics; Billerica, MA) operated in linear, positive mode at a laser frequency of 60 Hz (100 shots total). The spectra were calibrated using the protein Standard II from Bruker-Daltonics. Mass spectra were analyzed with the FLEX Analysis software (Bruker Daltonics).

### Size exclusion chromatography

The size and composition of purified mKate and mKate-IgGBP was analyzed by size exclusion chromatography on a Dionex FPLC equipped with a Superdex 75 column (GE Healthcare) as previously described [20]. Briefly, the column was operated at a flow rate of 0.5 mL/min in D-PBS and the eluate was monitored at 280 nm. Column calibration was performed using gel filtration standards (Bio-Rad; Hercules, CA) containing bovine thyroglobulin (670 kDa), bovine γ-globulin (158 kDa), chicken ovalbumin (44 kDa), horse myoglobin (17 kDa), and vitamin B_12_ (1.35 kDa) and plotted as Log_10_ molecular weight, in kilo-daltons, versus retention time. The standard curve was used to estimate the molecular weight of purified mKate and mKate-IgGBP.

### Competition ELISA to evaluate IgG binding to mKate-IgGBP

The ability of hIgG1, mIgG1, mIgG2a, mIgG2b, and rIgG2b to bind mKate-IgGBP was evaluated using a competition-based enzyme-linked immunosorption assay (ELISA). The wells of a Costar 3690 plate were coated with 70 µL of a 10 ng/mL solution of mKate, mKate-IgGBP, or SpA in D-PBS overnight at 4°C. Wells were washed 3 times with 150 µL of PBS-T (PBS, 0.05% Tween-20) and blocked for 1 hr at room temperature with 200 µL of a 3% BSA solution in PBS-T. A 1∶4000 dilution of donkey anti-chicken IgY IgG-HRP (dIgG-HRP) was co-incubated with increasing concentrations of IgG in blocking buffer on mKate-IgGBP coated wells for 1 hr at room temperature. We used commercially available dIgG-HRP as the detection reagent since the antigen specificity (chicken IgY) is irrelevant in this assay and therefore does not interfere with competition and dIgG-HRP binds specifically to mKate-IgGBP (Figure S2b in File S1). After 6 washes with PBS-T, bound IgG-HRP was detected by addition of the HRP substrate TMB (70 µL per well). Color was developed for 5 minutes, quenched with a 0.5 M sulfuric acid solution (70 µL per well) and absorbance was measured at 450 nm with blank subtraction at 550 nm. The absorbance for each test protein was normalized to the maximum absorbance observed for dIgG-HRP binding in the absence of competitor. All incubations were done in triplicate and data is represented as mean ± SD. The half-maximal inhibitory concentration (IC_50_) for each experimental protein was determined by fitting the data to a one-site LogIC_50_ model in Prism.

Dose-dependent binding of dIgG-HRP to mKate, mKate-IgGBP, or SpA coated plates was as described above with serial dilutions of dIgG-HRP in the absence of a competitor.

### Affinity measurements by surface plasmon resonance

SPR measurements were obtained using a BIAcore T100 instrument (BIAcore Inc.; Piscataway, NJ). Human IgG1, mouse IgG1, and rat IgG2b were captured on a CM5 sensor chip by amine coupling at pH 5 to a final immobilization density of ∼1500, ∼2000, and ∼2100 resonance units (RU), respectively. Un-reacted sites were blocked with 1 M ethanolamine. A control flow cell without immobilized IgG was prepared for reference subtraction. Dilutions of mKate, mKate-IgGBP, and SpA in running buffer [D-PBS, 50 mM Hepes, 0.05% Tween 20, pH 7.4 (PBS-T, pH 7.4) or D-PBS, 50 mM MES, 0.05% Tween 20, pH 6 (PBS-T, pH 6)] were injected over the chip for 225 sec followed by a 225 sec dissociation in running buffer. The chip was regenerated with a 30 sec injection of 10 mM glycine, pH 1.5 and two 45 sec injections of PBS-T, pH 7.4 or pH 6. The flow rate used for all methods was 30 µL/min. Binding kinetics and affinities were derived by analysis of the generated sensograms using the Biacore T100 evaluation software. Sensograms were fit to a 1∶1 binding model included in the evaluation software for derivation of binding kinetics and affinity (K_D_).

### FACS based FcRn competition assay

The ability of mKate, mKate-IgGBP, and hIgG1 to compete with labeled hIgG1 for human FcRn binding and subsequent accumulation in MDCK hFcRn-EYFP/hβ_2_m cells was quantified by FACS. We previously used this cell model to quantify FcRn binding by FACS and evaluate endocytosis, recycling, and transcytosis of various human FcRn ligands [17]. Cells were seeded (∼30,000 per well) in a 96-well plate and cultured for 24–48 hrs. Cells were washed twice in binding buffer [HBSS, 1% ovalbumin, 50 mM MES, pH 6] and co-incubated with 1 µM of hIgG1-TAMRA and increasing concentrations of either mKate, mKate-IgGBP, or unlabeled hIgG1 in binding buffer for 1 hr at 37°C, pH 6. Cells were washed three times with cold binding buffer to remove unbound protein, trypsinized, and analyzed on a FACS Array cell sorter (BD Biosciences; San Jose, CA). The mean fluorescent intensities (MFI) for each data point were derived after gating for live and EYFP positive cells. The MFI for each data point within a test protein (e.g. mKate-IgGBP) was normalized to the maximum MFI observed for labeled hIgG1 in the absence of competitor. All incubations were done in triplicate and data is represented as mean ± SD. The half-maximal inhibitory concentration (IC_50_) for each experimental protein was determined by fitting the data to a one-site LogIC_50_ model in Prism.

### Mouse plasma IgG concentration ELISA

The concentration of IgG in the plasma of 6–8 week old C57BL/6J, hFcRn Tg, and FcRn^−/−^ mice was determined by ELISA. Blood samples were collected into heparinized tubes through submandibular cheek pouch bleeds. Blood was centrifuged at 6,000 rpm for 6 min and the plasma was stored at –80°C until use. Quantification of mouse IgG in plasma was determined using the Mouse IgG ELISA Quantitation Set (Bethyl Laboratories, Inc.; Montgomery, TX) following the manufactures recommended protocol. C57BL/6J plasma was diluted 1∶2500 in sample buffer (D-PBS, 0.05% Tween 20, 3% BSA) whereas hFcRn Tg and FcRn^−/−^ plasma was diluted 1∶500 in sample buffer to achieve absorbance values within the range of the standard curve. A 1∶40000 dilution of the HRP detection antibody in sample buffer was used for this assay.

### Plasma clearance in mice

The plasma clearance of mKate, mKate-IgGBP, mIgG1, and hIgG1 were evaluated in wild type C57BL/6J and/or hFcRn Tg mice. Mouse IgG1 was labeled with amine reactive 5-Carboxytetramethylrhodamine (5-TAMRA, Ex/Em: 546/579) and hIgG1 was labeled with amine reactive AlexaFluor 647 (Ex/Em: 651/672) at a ratio ∼2–3 mol fluorophore per mol IgG1 to enable detection in mouse plasma via fluorescence. mKate and mKate-IgGBP plasma clearance was assayed directly based on the intrinsic far-red fluorescent properties of mKate (Ex/Em: 588/635). Mice (n = 9 per group) received a 10 mg/kg intravenous (i.v.) tail vein injection of mKate, mKate-IgGBP, or labeled IgG1 in 200 µL D-PBS and blood samples were collected into heparinized tubes through submandibular cheek pouch bleeds. Blood was centrifuged at 6,000 rpm for 6 min and the plasma was diluted 1∶10 into D-PBS and assayed by fluorometry on a Spex Fluorolog fluorometer (Horiba Jobin Yvon; Edison, NJ) with 5 nm excitation and emission slits. All plasma samples were normalized to the maximum fluorescence observed in the first bleed 5 min after injection and plotted as % injected dose (%ID) versus time. The α- and β- phase half-life of mKate-IgGBP and hIgG1 were calculated by fitting the plasma clearance curves (%ID vs. time) to a 2-compartment PK model, Y = C_0_(e^k1*X^)+C_1_(e^k2*X^), using Prism 5 (GraphPad Software; La Jolla, CA). The half-life of mKate was calculated by separately fitting the %ID vs. time data to a semilog line model, Y = 10^(k*X+Y0)^, in Prism 5.

The plasma clearance of mKate-IgGBP co-administered with hIgG1-Alexa was determined in hFcRn Tg mice. mKate-IgGBP and hIgG1-Alexa were pre-mixed in a 1∶1 mol ratio of mKate-IgGBP:hIgG1-Alexa in D-PBS. Mice (n = 9 per group) received a 200 µL i.v. tail vein injection of the mixture corresponding to a 3 mg/kg dose of mKate-IgGBP and a 15 mg/kg dose of hIgG1-Alexa. Blood was collected and assayed as described above to determine the clearance of both mKate-IgGBP and hIgG1-Alexa in a co-administration setting.

### Statistical analysis

Comparison between multiple groups was analyzed for statistical significance using a one-way ANOVA and Bonferroni post-test. All statistical analysis was performed in Prism 5 (GraphPad Software) on untransformed data.

## Results and Discussion

### IgGBP Fusion Protein Expression and Characterization

**Figure 2:**
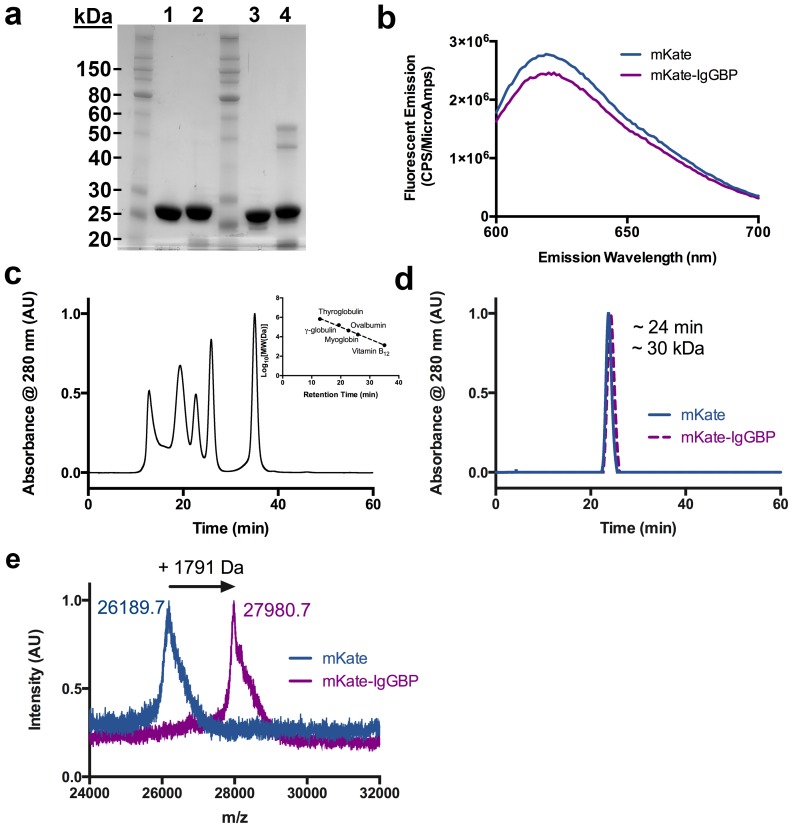
Characterization of mKate and mKate-IgGBP fusion. (a) SDS-PAGE analysis of purified mKate (lanes 1, 3) and mKate-IgGBP (lanes 2, 4) under reducing (lanes 1, 2) and non-reducing conditions (lanes 3, 4). 7.5 ug of protein was loaded in each lane. (b) Fluorescence emission spectra comparing equal molar concentrations (500 nM in D-PBS) of mKate and mKate-IgGBP. (c) Size exclusion chromatography standards and associated standard curve. The standards include thyroglobulin 670 kDa, gamma-globulin 158 kDa, ovalbumin 44 kDa, myoglobin 17 kDa, and vitamin B12 1.35 kDa. (d) Size exclusion chromatogram of mKate and mKate-IgGBP with retention time and calculated molecular weight. (e) MALDI-TOF analysis of mKate and mKate-IgGBP intact mass indicating a shift in molecular weight corresponding to the expected mass of the added flexible linker and IgGBP sequence.

To test the hypothesis that proteins modified with an IgG binding peptide results in half-life extension, we fused the 13 amino acid FcIII sequence [16] separated by a short, flexible Gly_4_Ser linker to the C-terminus of a model fluorescent protein, monomeric Katushka [21] (mKate) (Figure 1a). The mKate-IgGBP fusion protein was expressed in the soluble fraction of *E. coli* and after purification to near homogeneity retains its fluorescence emission properties (Figure 2a,b). IgGBP fusion does not alter migration by SDS-PAGE (Figure 2a) or size exclusion chromatography under non-denaturing conditions (Figure 2c,d), indicating that IgGBP fusion does not increase the apparent molecular weight. MALDI-TOF MS analysis confirms the addition of a single IgGBP to the C-terminus of mKate (Figure 2e).

### Competition ELISA to Evaluate IgG binding to mKate-IgGBP

**Figure 3:**
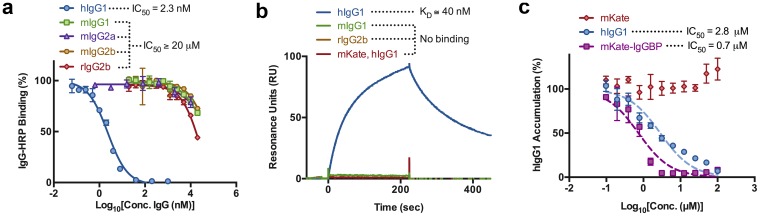
IgGBP fusion results in specific and high affinity binding to human IgG1 at a site overlapping the FcRn. (**a**) Competition ELISA between dIgG-HRP and unlabeled IgGs binding to mKate-IgGBP coated plates. (**b**) Sensograms demonstrating mKate-IgGBP (100 nM) binding to immobilized hIgG1 but not mIgG1 or rIgG2b. Unmodified mKate lacks binding to immobilized hIgG1 by SPR. (**c**) Competition of labeled hIgG1 accumulation in MDCK hFcRn-EYFP/hβ_2_m cells at pH 6 by unlabeled hIgG1, mKate-IgGBP, and mKate. MDCK hFcRn-EYFP/hβ_2_m cells were co-incubated with 1 µM labeled hIgG1-TAMRA and increasing concentrations of unlabeled hIgG1, mKate-IgGBP, and mKate for 1 hr at 37°C and analyzed by FACS as described in the methods section. The mean fluorescent intensity (MFI) of each test protein was normalized to the average MFI of hIgG1-TAMRA accumulation in MDCK hFcRn-EYFP/hβ_2_m cells in the absence of unlabeled competitor and plotted as the % of hIgG1-TARMA accumulation as a function of competitor concentration. The data shown are the mean and error bars indicate s.d.

We determined the ability of IgG from various species to bind the target proteins using a competition based ELISA in which either mKate or mKate-IgGBP are absorbed to a 96-well plate and donkey IgG-HRP (dIgG-HRP) is used as the detection reagent (Figure S1a in File S1). Donkey IgG-HRP exhibits dilution dependent binding to mKate-IgGBP but not mKate coated plates, indicating that dIgG-HRP binding is specific for the IgGBP sequence (Figure S1b in File S1). Human IgG1 potently inhibits dIgG-HRP binding to mKate-IgGBP with an IC_50_ of ∼2.4 nM whereas mouse IgG1 (mIgG1), mIgG2a, mIgG2b, and rat IgG2b (rIgG2b) are weak inhibitors (IC_50_>20 µM) (Figure 3a), suggesting that mKate-IgGBP binding to IgG is species-dependent with specificity for human and donkey but not mouse or rat IgG.

Species specificity among alternative IgG-Fc binding proteins, including SpA and FcRn, is commonly observed [22], [23]. SpA binds human IgG1, IgG2 and IgG4 as well as mouse IgG2a and IgG2b but lacks binding to mouse IgG1. We confirmed this species specificity of SpA using the competitive ELISA. Donkey IgG-HRP exhibits dilution dependent binding to SpA coated plates (Figure S1b in File S1) and is inhibited by co-incubation with excess human IgG1 (IC_50_ ∼22 nM), mIgG2a (IC_50_ ∼61 nM), and mIgG2b (IC_50_ ∼54 nM), but not mIgG1 (Figure S2 in File S1). These results confirm the known specificity of SpA for IgG [23] and indicate that the lack of competition between dIgG-HRP and mouse IgG isotypes for binding mKate-IgGBP is due to the IgGBP specificity and not the reagents used in the assay.

Our results contrast that of Sakamoto et al. who demonstrated FcIII binding to mouse IgG using a single point (concentration) ELISA [24]. It is difficult to determine with confidence the reason for the difference in reactivity observed between our study and that of Sakamoto et al. as the assay formats differ; however, a probable explanation is the use of peptide-streptavidin (SA) tetramers by Sakamoto et al. to detect binding to mouse IgG coated plates. SA tetramer staining is a common technique used to detect low affinity interactions by increasing the avidity of the binding ligand. Therefore, it is possible that FcIII reacts weakly with mouse IgG but is not detectable under the assay conditions we used in this study.

### Binding Kinetics between IgG and mKate-IgGBP

To further confirm species specificity and quantify binding we measured binding kinetics by surface plasmon resonance (SPR). mKate-IgGBP binds immobilized hIgG1 with a K_D_ of ∼40 nM and ∼20 nM at pH 7.4 and pH 6, respectively, whereas unmodified mKate does not bind to hIgG1 (Figure 3b, Figure S3a,d,e in File S1, and Table 1). mKate-IgGBP and SpA lack binding to mIgG1 and rIgG2b at concentrations up to 500 nM (Figure 3b, Figure S3b,c in File S1, and Figure S4b,c in File S1) confirming the species specificity of mKate-IgGBP for hIgG1 observed by ELISA. The affinity of mKate-IgGBP for hIgG1 is only slightly lower than SpA (Figure S4a,b in File S1 and Table 1) despite the significant difference in molecular weight of the two IgG binding ligands.

**Table 1:** Binding kinetics of mKate, mKate-IgGBP, and SpA to hIgG1, mIgG1, and rIgG2b determined by SPR.

Molecule	IgG Species	k_a_ pH 7.4 (10^5^/Ms)	k_d_ pH 7.4 (10^−3^/s)	K_D_* pH 7.4 (nM)	k_a_ pH 6 (10^5^/Ms)	k_d_ pH 6 (10^−3^/s)	K_D_* pH 6 (nM)
mKate	Human, Mouse, Rat	–	–	**No binding**	–	–	**No binding**
mKate-IgGBP	Human	1.1	4.2	**40**	2.7	5.3	**19**
mKate-IgGBP	Mouse, Rat	–	–	**No binding**	–	–	**No binding**
SpA	Human	1.3	23	**2**	0.8	1.2	**16**
SpA	Mouse, Rat	–	–	**No binding**	–	–	**No binding**

(*) Data were fit to a 1∶1 kinetic binding model for derivation of K_D_.

### Inhibition of hIgG1 binding to FcRn by mKate-IgGBP

The crystal structure of FcIII in complex with human IgG-Fc [16] indicates a binding site overlapping that of human FcRn (hFcRn). Therefore, we determined the ability of mKate-IgGBP to inhibit hIgG1 binding to hFcRn using an *in vitro* cell-based competition assay. We previously used this cell model to quantify FcRn binding by FACS and evaluate endocytosis, recycling, and transcytosis of various human FcRn ligands [17]. Human IgG1-TAMRA is endocytosed specifically by hFcRn-EYFP expressed in MDCK cells when incubated at pH 6 and 37°C [17]. Therefore, we utilized this cell model to quantify the ability of mKate-IgGBP to inhibit hIgG1-TAMRA binding to the FcRn. Because the assay conditions permit FcRn-dependent endocytosis of hIgG1-TAMRA and not just surface binding, the data is presented as “% hIgG1 accumulation” instead of “% hIgG1 binding.”

Unlabeled hIgG1 inhibits the cellular accumulation of labeled hIgG1 with an IC_50_ of ∼2.8 µM (Figure 3c). mKate-IgGBP also inhibits the cellular accumulation of labeled hIgG1 with increased potency (IC_50_≅0.7 µM), whereas unmodified mKate has no affect on the accumulation of hIgG1 at concentrations up to 100 µM (Figure 3c). Therefore, in addition to extending the half-life of protein cargo, the IgGBP also has the potential for use as an antagonist of the IgG-FcRn interaction to treat IgG-mediated autoimmune diseases, as can alternative inhibitors of the IgG-FcRn axis [25]–[28], however this would need to be validated experimentally.

### *In vivo* fate of mKate-IgGBP in mice

**Figure 4:**
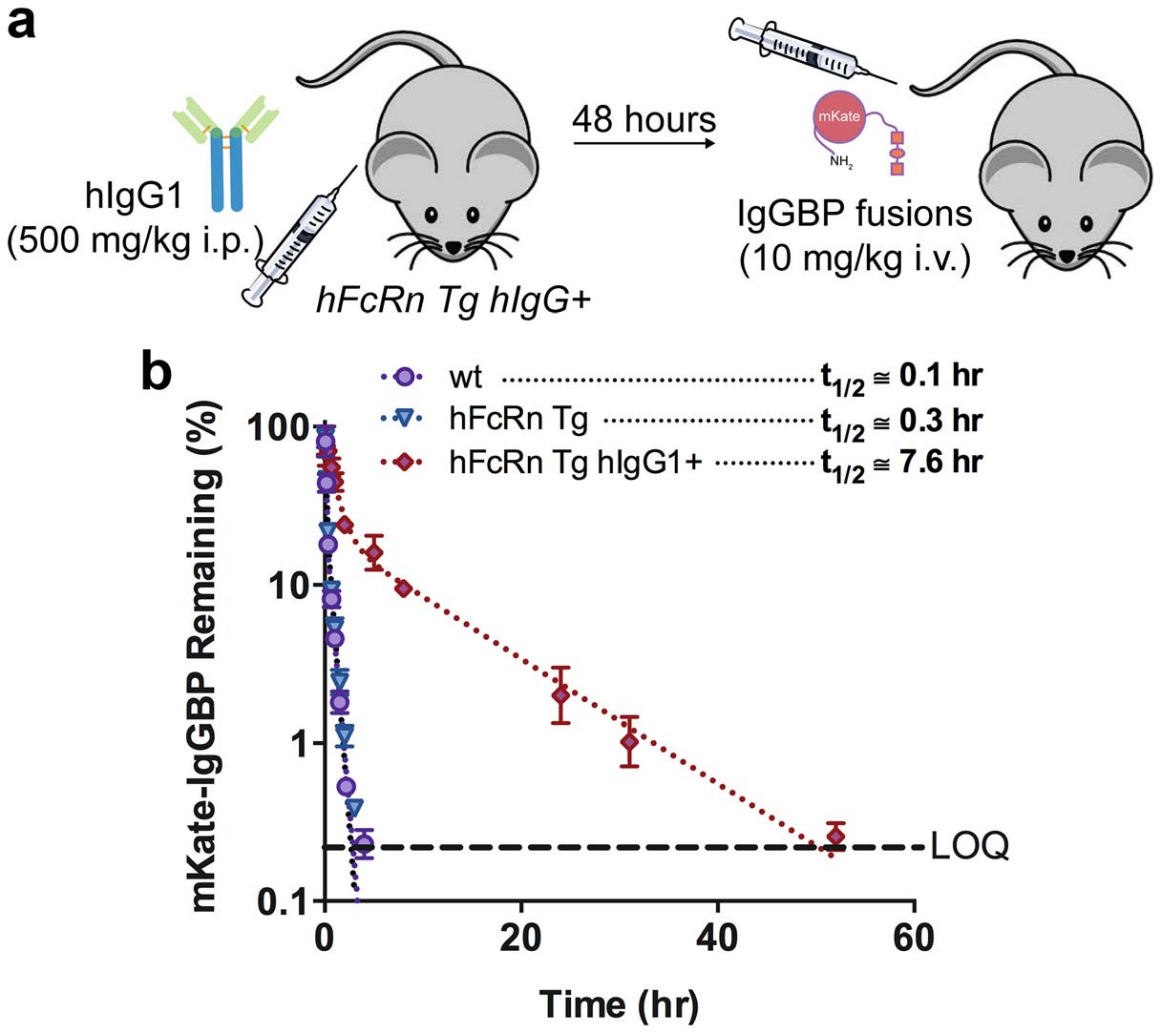
IgGBP fusion extends mKate half-life in hFcRn Tg hIgG+ mice. (**a**) Schematic of the hFcRn Tg hIgG1+ mouse model. hFcRn Tg mice were dosed i.p. with 500 mg/kg of recombinant hIgG1 48 hours prior to injection of mKate-IgGBP. (**b**) Clearance of mKate-IgGBP in wild-type (purple circles), hFcRn Tg (blue triangle), and hFcRn Tg hIgG1+ (red diamond) mice dosed i.v. at 10 mg/kg via the tail vein as a single agent. The % mKate-IgGBP remaining was calculated by normalizing the fluorescent emission at all time points to the maximum value observed in the first bleed 5 min after protein injection. Dashed lines represent the data fit to a 2-compartment PK model in Prism and the β-phase half-life shown in the figure was calculated as described in the Methods section. The data shown are the mean (n = 3 bleeds per time point) and error bars indicate s.d.

**Figure 5:**
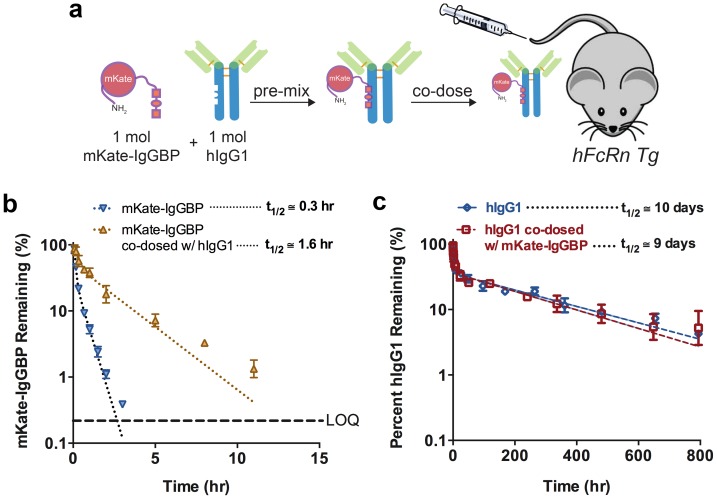
IgGBP fusion extends mKate half-life in hFcRn Tg mice when co-administered as a 1∶1 mol mixture with hIgG1 without altering hIgG1 clearance. (**a**) Schematic of the co-administration scheme. In this experiment, human FcRn Tg mice were not pre-dosed with exogenous hIgG1. Instead mKate-IgGBP and hIgG1 were pre-mixed in a 1∶1 mol ratio and co-injected via the tail vein. (**b**) Clearance of mKate-IgGBP in hFcRn Tg mice dosed alone (blue triangles) or co-dosed at a 1∶1 mol mixture with hIgG1 (yellow triangles). The % mKate-IgGBP remaining was calculated by normalizing the fluorescent emission at all time points to the maximum value observed in the first bleed 5 min after protein injection. (**c**) Clearance of labeled human IgG1 in hFcRn Tg mice dosed as a single agent via the tail vein (blue triangles) compared to the clearance of labeled hIgG1 co-administered as a 1∶1 mol mixture with mKate-IgGBP was measured to determine if bound mKate-IgGBP alters the eliminate profile of hIgG1 (red squares). The % hIgG1 remaining was calculated by normalizing the fluorescent emission at all time points to the maximum value observed in the first bleed 5 min after protein injection. Dashed lines in each panel represent the data fit to a 2-compartment PK model in Prism and the β-phase half-life shown in the figure was calculated as described in the Methods section. The data shown in each panel are the mean (n = 3 bleeds per time point) and error bars indicate s.d.

Given that mKate-IgGBP binds to hIgG1 with high affinity *in vitro* and that IgG is highly concentrated in serum with a remarkably long half-life, we determined the *in vivo* fate of mKate-IgGBP. The specificity of mKate-IgGBP for human IgG necessitates the use of a mouse model that can support high concentrations of hIgG in serum. Therefore, we utilized a human FcRn transgenic mouse (hFcRn Tg) model developed by Derry Roopenian and colleagues at the Jackson Laboratory [19], [29]. hFcRn Tg mice have low endogenous mouse plasma IgG levels and accelerated mouse IgG clearance (Figure S5 and Figure S6 in File S1) due to the weak interaction between human FcRn and mouse IgG isotypes [22]. We “reconstituted” the plasma of hFcRn Tg mice with a high dose (500 mg/kg) of recombinant hIgG1, termed hFcRn Tg hIgG+ mice, 48 hrs prior to injection of the experimental proteins (Figure 4a). After the initial distribution phase, hIgG1 has a long terminal plasma half-life of ∼10 days in the hFcRn Tg mouse model (Figure 5c), which makes it suitable for determining the PK of hIgG1 specific ligands.

In hFcRn Tg mice lacking exogenous hIgG1, unmodified mKate and mKate-IgGBP are rapidly eliminated from circulation (t_1/2_ ∼5–10 min) due to their small size (∼25 kDa) and lack of available hIgG1 to bind *in vivo* (Figure 4b and Figure S7 in File S1). mKate-IgGBP is also rapidly eliminated from wild type C57BL/6J mice despite the high plasma concentrations of mouse IgG (Figure 4b and Figure S5 in File S1) consistent with the inability of mKate-IgGBP to bind mouse IgG isotypes *in vitro*. In contrast, we observed an ∼75-fold increase in half-life of mKate-IgGBP in hFcRn Tg mice pre-dosed with a high concentration of hIgG1 (t_1/2_ ∼8 hr; Figure 4b). This supports the hypothesis that engineering proteins to interact with serum IgG through a short, C-terminal peptide extension enables prolonged blood circulation. Half-life extension by IgGBP fusion is similar to that obtained with SpA and/or SpG domain fusions [13] despite the significant difference in size of the IgG binding modules (13 versus 50–60 amino acids). The termini of a number of therapeutically relevant protein drugs and imaging agents are amenable for genetic modification [30]; therefore, IgGBP fusion may be a general strategy to improve protein half-life with minimal modification size and complexity.

We also evaluated the clearance of labeled hIgG1 co-dosed in a 1∶1 mol ratio with mKate-IgGBP in hFcRn Tg mice lacking exogenous hIgG1, e.g. not pretreated with 500 mg/kg hIgG1 (Figure 5a). The plasma clearance of hIgG1 are similar in the single agent and co-dose conditions indicating that mKate-IgGBP does not alter the elimination of hIgG1 under the dosing conditions used in this study (Figure 5c). However, in the co-dose setting the half-life of mKate-IgGBP is extended ∼16-fold compared to mKate-IgGBP dosed without hIgG1 (Figure 5b). The more rapid plasma clearance of mKate-IgGBP when co-dosed with hIgG1 compared to dosing as a single agent to hFcRn Tg hIgG+ mice is expected given the much higher concentrations of hIgG1 in the hFcRn Tg hIgG+ mouse; the high hIgG1 concentration increases the frequency of a productive mKate-IgGBP and hIgG1 interaction. Thus, both single agent and co-administration of mKate-IgGBP results in a significant extension of protein half-life.

## Conclusions

We describe a simple approach to substantially improve protein half-life without the necessity to increase molecular weight by engineering serum IgG binding using a low molecular weight IgG-Fc binding peptide fused to the C-terminus of a model protein. Such IgGBP fusion proteins should be easier to manufacture than Fc- and albumin fusions. Further increases in IgGBP affinity for Fc at the interface between its C_H_2 and C_H_3 domains and/or the generation of high affinity ligands to other binding sites on IgG may enable IgG-like half-lives of small recombinant proteins.

The half-life extensions obtained by IgGBP fusion are similar to that reported by Mark Dennis and co-workers who identified peptides that bind with high affinity to serum albumin and constructed albumin binding peptide-protein fusions to increase the serum half-life of Fab fragments. Our results provide additional evidence to support the concept of targeting abundant serum proteins, such as IgG and albumin, to increase protein half-life. The IgGBP fusion approach promoted herein buttresses the foundation for this half-life extension strategy that may improve the drug-like properties of numerous rapidly eliminated therapeutic proteins, macromolecule drugs, or drug carriers.

## Supporting Information

File S1 **Figures S1–S7.** Figure S1. IgG and mKate-IgGBP competition ELISA. (a) Schematic depicting the competition ELISA format for detecting IgG binding to mKate-IgGBP. Donkey IgG-HRP binds mKate-IgGBP coated plates. Co-incubation with a competitor blocks donkey IgG-HRP binding to mKate-IgGBP. (b) Dose-dependent binding of donkey IgG-HRP to mKate-IgGBP and SpA but not mKate coated plates. Data shown are the mean (n = 3) and error bars indicate s.d. Figure S2. Competition ELISA between donkey IgG-HRP and unlabeled IgGs binding to SpA coated plates. The data shown are the mean (n = 3) and error bars indicate s.d. Solid lines represent data fit to a one-site log IC_50_ model in Prism. Figure S3. SPR sensograms of mKate and mKate-IgGBP binding to immobilized IgG. Increasing concentrations of mKate-IgGBP were injected over immobilized hIgG1 (a), mIgG1 (b), and rIgG2b (c) at pH 7.4 or hIgG1 at pH 6 (d) as described in the Supplementary Methods. Unmodified mKate does not bind immobilized mouse, rat, or human IgG at concentrations up to 5000 nM (e). The resulting sensograms in (a) and (d) were fit to a 1∶1 kinetic binding model for derivation of K_D_. All data were baseline-adjusted and reference cell-subtracted. Figure S4. SPR sensograms of SpA binding to immobilized IgG. Increasing concentrations of SpA were injected over immobilized hIgG1 (a,d), mIgG1 (b,e), and rIgG2b (c,f) at pH 7.4 (a–c) or pH 6 (d–f) as described in the Supplementary Methods. The resulting sensograms in (a) and (d) were fit to a 1∶1 kinetic binding model for derivation of K_D_. All data were baseline-adjusted and reference cell-subtracted. Figure S5. Mouse plasma IgG levels in wild-type C57BL/6J mice, hFcRn Tg mice, and FcRn-null mice. The concentration of mouse IgG in the plasma of 6–8 week old C57BL/6J (n = 4), hFcRn Tg (n = 4), and FcRn^−/−^ mice (n = 3) was determined by ELISA as described in the Supplementary Methods. The plasma IgG concentration in C57BL/6J is significantly higher (p<0.005) than in hFcRn Tg (Tg32 homoz.) and FcRn-null mice. No statistical difference between hFcRn Tg and FcRn^−/−^ plasma IgG concentration is observed. Figure S6. Plasma clearance of mouse IgG1 in wild-type C57BL/6J and hFcRn Tg mice. (a) Labeled mouse IgG1 was dosed i.v. at 10 mg/kg via the tail vein to 7–8 week old wild-type C57BL/6J mice (purple diamonds) or hFcRn Tg mice (green triangles). Blood was collected at various time points into heparized tubes and the plasma clearance of labeled mIgG1 was determined via fluorometry. The % mIgG1 remaining was calculated by normalizing the fluorescent emission at all time points to the maximum value observed in the first bleed 5 min after injection of labeled mIgG1. Dashed lines represent the data fit to a 2-compartment PK model in Prism and the β-phase half-life shown in the figure was calculated as described in the Methods section. The data shown in each panel are the mean (n = 3 bleeds per time point) and error bars indicate s.d. Figure S7. Plasma clearance of unmodified mKate in wild-type C57BL/6J and hFcRn Tg mice. mKate was dosed i.v. at 10 mg/kg via the tail vein. Blood was collected at various time points into heparized tubes and the plasma clearance of labeled protein was determined via fluorometry based on the intrinsic far-red fluorescent properties of mKate. The % mKate remaining was calculated by normalizing the fluorescent emission at all time points to the maximum value observed in the first bleed 5 min after protein injection. Dashed lines represent the data fit to a semi-log line model in Prism and the half-life shown in the figure was calculated as described in the Supplementary Methods. The data shown in each panel are the mean (n = 3 bleeds per time point) and error bars indicate s.d. LOQ, limit of quantification.
(PDF)
