# Supplementary material for: Fusion of a Short Peptide that Binds Immunoglobulin G to a Recombinant Protein Substantially Increases Its Plasma Half-Life in Mice
Source: PLoS One. 2014 Jul 24;9(7):e102566. doi: 10.1371/journal.pone.0102566 (PMC4109916; doi:10.1371/journal.pone.0102566)
Supplement: File S1 — Figures S1–S7. Figure S1. IgG and mKate-IgGBP competition ELISA. (a) Schematic depicting the competition ELISA format for detecting IgG binding to mKate-IgGBP. Donkey IgG-HRP binds mKate-IgGBP coated plates. Co-incubation with a competitor blocks donkey IgG-HRP binding to mKate-IgGBP. (b) Dose-dependent binding of donkey IgG-HRP to mKate-IgGBP and SpA but not mKate coated plates. Data shown are the mean (n = 3) and error bars indicate s.d. Figure S2. Competition ELISA between donkey IgG-HRP and unlabeled IgGs binding to SpA coated plates. The data shown are the mean (n = 3) and error bars indicate s.d. Solid lines represent data fit to a one-site log IC50 model in Prism. Figure S3. SPR sensograms of mKate and mKate-IgGBP binding to immobilized IgG. Increasing concentrations of mKate-IgGBP were injected over immobilized hIgG1 (a), mIgG1 (b), and rIgG2b (c) at pH 7.4 or hIgG1 at pH 6 (d) as described in the Supplementary Methods. Unmodified mKate does not bind immobilized mouse, rat, or human IgG at concentrations up to 5000 nM (e). The resulting sensograms in (a) and (d) were fit to a 1∶1 kinetic binding model for derivation of KD. All data were baseline-adjusted and reference cell-subtracted. Figure S4. SPR sensograms of SpA binding to immobilized IgG. Increasing concentrations of SpA were injected over immobilized hIgG1 (a,d), mIgG1 (b,e), and rIgG2b (c,f) at pH 7.4 (a–c) or pH 6 (d–f) as described in the Supplementary Methods. The resulting sensograms in (a) and (d) were fit to a 1∶1 kinetic binding model for derivation of KD. All data were baseline-adjusted and reference cell-subtracted. Figure S5. Mouse plasma IgG levels in wild-type C57BL/6J mice, hFcRn Tg mice, and FcRn-null mice. The concentration of mouse IgG in the plasma of 6–8 week old C57BL/6J (n = 4), hFcRn Tg (n = 4), and FcRn−/− mice (n = 3) was determined by ELISA as described in the Supplementary Methods. The plasma IgG concentration in C57BL/6J is significantly higher (p<0.005) than in hFcRn Tg (Tg [file pone.0102566.s001.pdf]

Supplementary Information

Fusion of a Short Peptide that Binds Immunoglobulin G to a Recombinant Protein Substantially Increases its Plasma Half-life in Mice

Sockolosky JT, Kivimäe S, Szoka FC

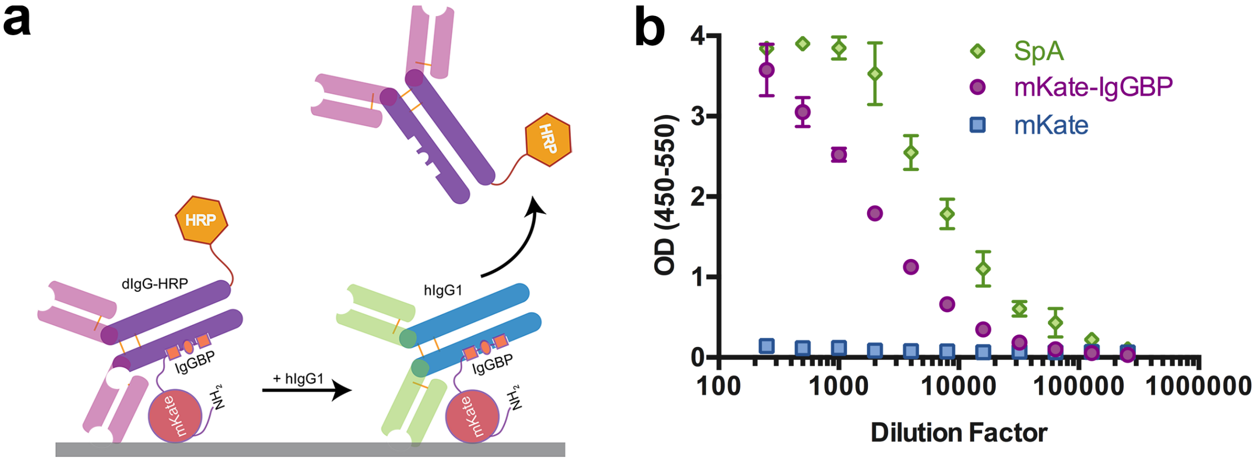

Supplementary Figure S1. IgG and mKate-IgGBP competition ELISA. (a) Schematic depicting the competition ELISA format for detecting IgG binding to mKate-IgGBP. Donkey IgG-HRP binds mKate-IgGBP coated plates. Co-incubation with a competitor blocks donkey IgG-HRP binding to mKate-IgGBP. (b) Dose-dependent binding of donkey IgG-HRP to mKate-IgGBP and SpA but not mKate coated plates. Data shown are the mean (n=3) and error bars indicate s.d.

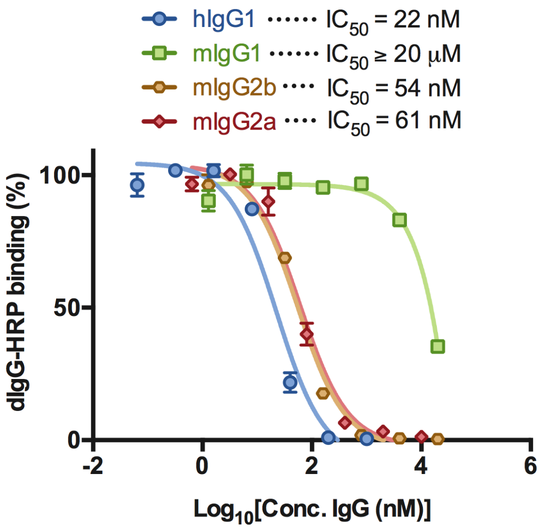

Supplementary Figure S2. Competition ELISA between donkey IgG-HRP and unlabeled IgGs binding to SpA coated plates. The data shown are the mean (n=3) and error bars indicate s.d. Solid lines represent data fit to a one-site log IC_{50} model in Prism.

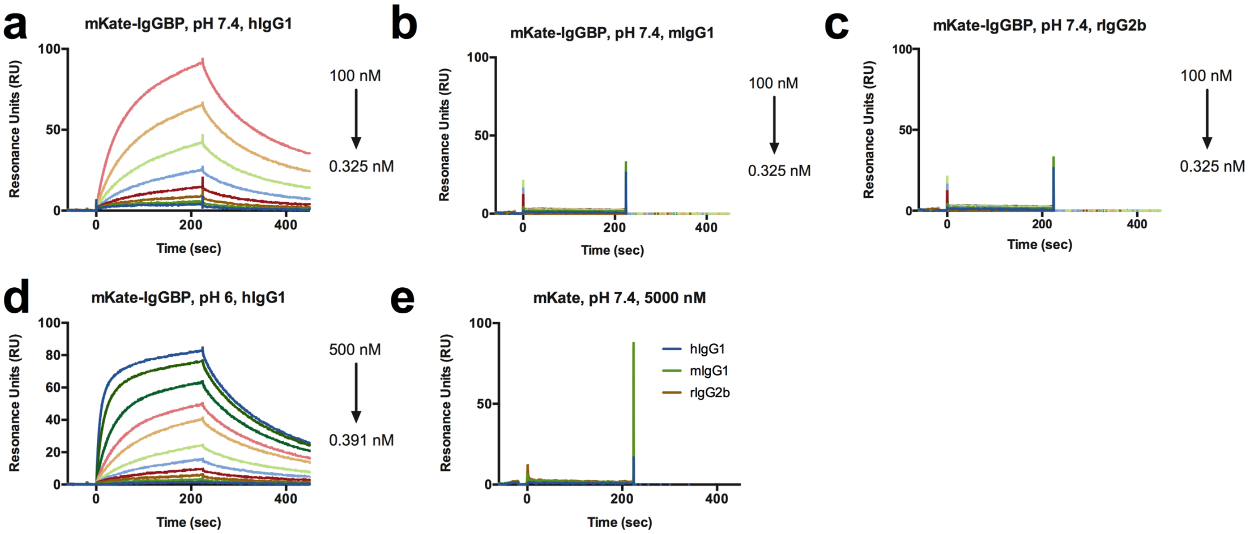

Supplementary Figure S3. SPR sensograms of mKate and mKate-IgGBP binding to immobilized IgG. Increasing concentrations of mKate-IgGBP were injected over immobilized hIgG1 (a), mIgG1 (b), and rIgG2b (c) at pH 7.4 or hIgG1 at pH 6 (d) as described in the Supplementary Methods. Unmodified mKate does not bind immobilized mouse, rat, or human IgG at concentrations up to 5000 nM (e). The resulting sensograms in (a) and (d) were fit to a 1:1 kinetic binding model for derivation of K_D. All data were baseline-adjusted and reference cell-subtracted.

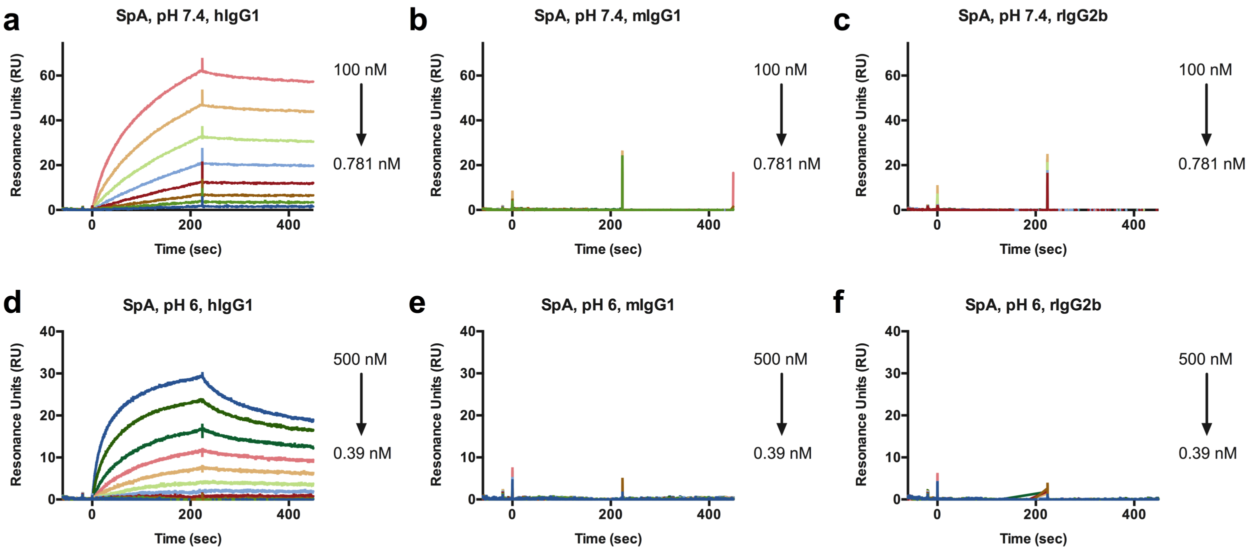

Supplementary Figure S4. SPR sensograms of SpA binding to immobilized IgG. Increasing concentrations of SpA were injected over immobilized hIgG1 (a,d), mIgG1 (b,e), and rIgG2b (c,f) at pH 7.4 (a-c) or pH 6 (d-f) as described in the Supplementary Methods. The resulting sensograms in (a) and (d) were fit to a 1:1 kinetic binding model for derivation of K_D. All data were baseline-adjusted and reference cell-subtracted.

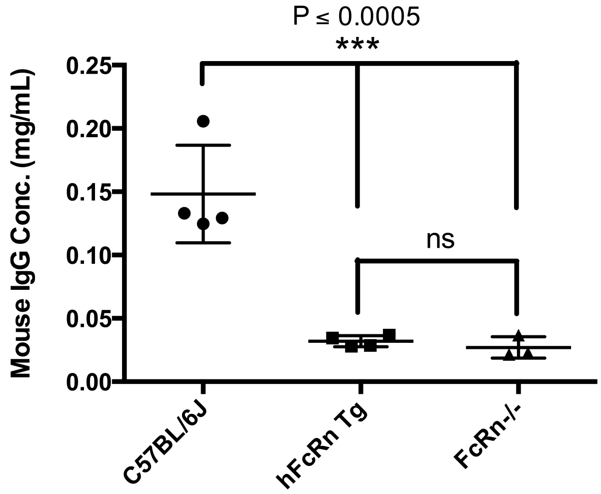

Supplementary Figure S5. Mouse plasma IgG levels in wild-type C57BL/6J mice, hFcRn Tg mice, and FcRn-null mice. The concentration of mouse IgG in the plasma of 6-8 week old C57BL/6J (n=4), hFcRn Tg (n=4), and $FcRn^{-/-}$ mice (n=3) was determined by ELISA as described in the Supplementary Methods. The plasma IgG concentration in C57BL/6J is significantly higher (p<0.005) than in hFcRn Tg (Tg32 homoz.) and FcRn-null mice. No statistical difference between hFcRn Tg and $FcRn^{-/-}$ plasma IgG concentration is observed.

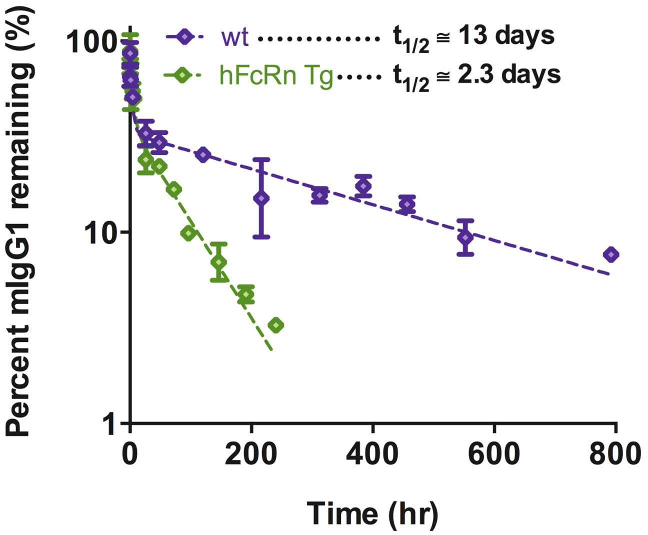

Supplementary Figure S6. Plasma clearance of mouse IgG1 in wild-type C57BL/6J and hFcRn Tg mice. (a) Labeled mouse IgG1 was dosed i.v. at 10 mg/kg via the tail vein to 7-8 week old wild-type C57BL/6J mice (purple diamonds) or hFcRn Tg mice (green triangles). Blood was collected at various time points into heparized tubes and the plasma clearance of labeled mIgG1 was determined via fluorometry. The % mIgG1 remaining was calculated by normalizing the fluorescent emission at all time points to the maximum value observed in the first bleed 5 min after injection of labeled mIgG1. Dashed lines represent the data fit to a 2-compartment PK model in Prism and the β-phase half-life shown in the figure was calculated as described in the Methods section. The data shown in each panel are the mean (n=3 bleeds per time point) and error bars indicate s.d.

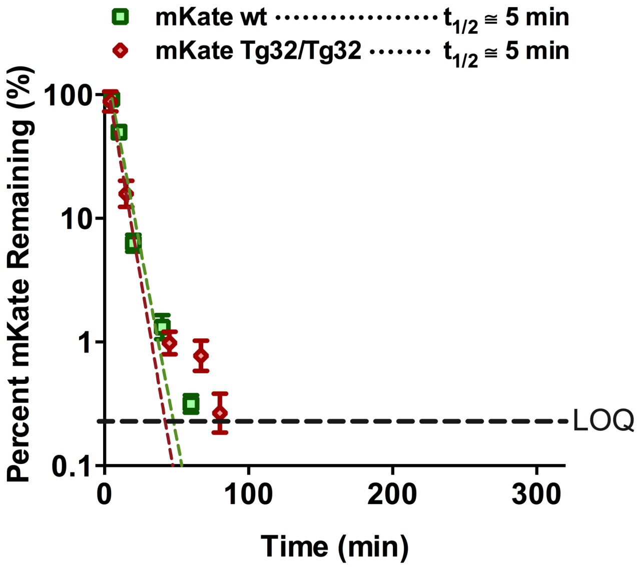

Supplementary Figure S7. Plasma clearance of unmodified mKate in wild-type C57BL/6J and hFcRn Tg mice. mKate was dosed i.v. at 10 mg/kg via the tail vein. Blood was collected at various time points into heparized tubes and the plasma clearance of labeled protein was determined via fluorometry based on the intrinsic far-red fluorescent properties of mKate. The % mKate remaining was calculated by normalizing the fluorescent emission at all time points to the maximum value observed in the first bleed 5 min after protein injection. Dashed lines represent the data fit to a semi-log line model in Prism and the half-life shown in the figure was calculated as described in the Supplementary Methods. The data shown in each panel are the mean (n=3 bleeds per time point) and error bars indicate s.d. LOQ, limit of quantification.
